# Unraveling *LMNA* Mutations in Metabolic Syndrome: Cellular Phenotype and Clinical Pitfalls

**DOI:** 10.3390/cells9020310

**Published:** 2020-01-28

**Authors:** Camille Desgrouas, Alice-Anaïs Varlet, Anne Dutour, Damien Galant, Françoise Merono, Nathalie Bonello-Palot, Patrice Bourgeois, Adèle Lasbleiz, Cathy Petitjean, Patricia Ancel, Nicolas Levy, Catherine Badens, Bénédicte Gaborit

**Affiliations:** 1 Aix Marseille Université, INSERM, MMG, 13005 Marseille, France; camille.desgrouas@univ-amu.fr (C.D.); alice-anais.varlet@univ-amu.fr (A.-A.V.); damien.galant@etu.univ-amu.fr (D.G.); francoise.merono@univ-amu.fr (F.M.); nathalie.bonello@ap-hm.fr (N.B.-P.); patrice.bourgeois@ap-hm.fr (P.B.); nicolas.levy@univ-amu.fr (N.L.); catherine.badens@univ-amu.fr (C.B.); 2 Aix Marseille Université, Laboratoire de Chimie Analytique, Faculté de Pharmacie, 13005 Marseille, France; 3 Aix Marseille Université, INSERM, INRAE, C2VN, 13005 Marseille, France; anne.dutour@ap-hm.fr (A.D.); adele.lasbleiz@ap-hm.fr (A.L.); patricia.ancel@univ-amu.fr (P.A.); 4 APHM, Endocrinology, Metabolic diseases and nutrition department, 13005 Marseille, France; petitjean.cathy@wanadoo.fr; 5 APHM, Hôpital de la Timone, Laboratoire de Génétique Moléculaire, 13005 Marseille, France

**Keywords:** dyslipidemia, metabolic syndrome, insulin resistance, lamin A/C, nuclear anomalies and premature senescence

## Abstract

This study details the clinical and cellular phenotypes associated with two missense heterozygous mutations in *LMNA*, c.1745G>T p.(Arg582Leu), and c.1892G>A p.(Gly631Asp), in two patients with early onset of diabetes mellitus, hypertriglyceridemia and non-alcoholic fatty liver disease. In these two patients, subcutaneous adipose tissue was persistent, at least on the abdomen, and the serum leptin level remained in the normal range. Cellular studies showed elevated nuclear anomalies, an accelerated senescence rate and a decrease of replication capacity in patient cells. In cellular models, the overexpression of mutated prelamin A phenocopied misshapen nuclei, while the partial reduction of lamin A expression in patient cells significantly improved nuclear morphology. Altogether, these results suggest a link between lamin A mutant expression and senescence associated phenotypes. Transcriptome analysis of the whole subcutaneous adipose tissue from the two patients and three controls, paired for age and sex using RNA sequencing, showed the up regulation of genes implicated in immunity and the down regulation of genes involved in development and cell differentiation in patient adipose tissue. Therefore, our results suggest that some mutations in *LMNA* are associated with severe metabolic phenotypes without subcutaneous lipoatrophy, and are associated with nuclear misshaping.

## 1. Introduction

Familial partial lipodystrophy (FPLD) is a genetic condition characterized by partial lipoatrophy and metabolic dysfunctions, such as insulin resistance and hypertriglyceridemia [1]. This condition predisposes to diabetes type 2, cardiovascular diseases and hepatic or pancreatic comorbidities. FPLD type 2 (FPLD2), commonly named Dunnigan syndrome, is the most frequent FPLD and results from *LMNA* mutations transmitted in a dominant pattern [2,3,4]. *LMNA* encodes for lamin A and C proteins as the result of an alternative splicing. These proteins are type V intermediate filaments located both in the nucleoplasm and at the nuclear lamina, a meshwork underlying the inner nuclear envelope. Lamins participate in the nuclear structure and in the chromatin-nuclear lamina connection, and consequently, to the set up and maintenance of gene expression pattern [5]. *LMNA*-related diseases, gathered in a group called laminopathies, display a large spectrum of clinical expression, from FPLD to extremely severe phenotypes, such as progeria syndrome and restrictive dermopathy. At the cellular level, and in vitro, all pathogenic *LMNA* mutations are associated with a decrease of cell proliferation rate, premature senescence, misshapen nuclei, and chromatin remodeling [6,7,8]. The level of these alterations is roughly correlated with the severity of clinical expression, cells from patients with progeria, and restrictive dermopathy presenting with the most severe cellular senescence.

Regarding FPLD2, mutations located on the residue 482 are responsible for 80% of the cases and are associated with the classical clinical form [9]. Interestingly, women are more often diagnosed than men due to the android appearance characterized by protruding and well-defined musculature [10,11]. They also suffer from more severe and more precocious metabolic perturbations and from gynecological disorders (polycystic ovarian syndrome, miscarriage, stillbirth) [12]. Among men, phenotypes are more attenuated and induce less severe metabolic complications [1].

Besides mutations on residue 482, other mutations located all along *LMNA* can also be responsible for FPLD2, but are often associated with atypical forms of the disease. Particularly, in some very rare cases, mutations have been associated with an attenuated phenotype of lipodystrophy without lipoatrophy and corresponding to severe metabolic syndrome (MS), a condition that shares common features with FPLD2, such as dyslipidemia, insulin resistance and central adiposity [10,13,14]. In these cases, the pathogenicity of the mutations must be firmly demonstrated to confirm that they are not simply silent polymorphisms, as could often be the case for missense mutations.

In this study, we report the detailed phenotypes of two patients with mutations in *LMNA* associated with severe metabolic syndrome and no lipoatrophy, together with the results of functional studies, to document the effect of these two rare heterozygous missense mutations on cell proliferation capacity and on nuclear phenotype.

## 2. Material and Methods

### 2.1. Patients

The two patients reported here were initially referred to the Endocrinology, Metabolic Diseases and Nutrition Department at the Assistance Publique des Hôpitaux de Marseille for insulin resistance diabetes care. One of them was part of a cohort described previously [14]. They gave their written informed consent for this study. The patients underwent a subcutaneous fat biopsy (needle aspiration) for RNA sequencing and adipocytes diameter quantification. DNA samples and primary fibroblast were available for both patients and stored at the certified Biological Resource Center (CRB) tissue, DNA and cells components (NF S96-900 & ISO 9001 v2015 Certification).

### 2.2. Plasmids and siRNAs

The two *LMNA* missenses mutations were introduced using the QuickChange II XL Site-Directed Mutagenesis Kit (Agilent technologies, Santa Clara, CA, USA) in the cDNA of prelamin A cloned in the pEGFP-C1 vector (Clontech Laboratories Inc., Mountain View, Santa Clara, CA, USA). Mutagenic primers were designed using the QuikChange Primer Design Program available online at https://www.genomics.agilent.com/primerDesignProgram.jsp (sequences available on request). The presence of the two mutations was checked by direct sequencing, according to standard procedures on ABI3500XL (Life Technologies, Carlsbad, CA, USA). Mutations were numbered according to the GenBank reference sequence NM_170707.3 and the Human Genome Variation Society recommendations (http://varnomen.hgvs.org/).

SiRNA treatment was directed against lamin A 3′UTR (3′-UUAUUGAAGAGAAUCUUU-5′, Eurogentec) and a scrambled siRNA (Eurogentec ref SR-CL005-005) was used as a negative control. In addition, 40,000 and 150,000 fibroblasts were seeded in coverslips (Lab-Tek, SPL Life Science, Gyeonggi-do, Korea) and 6-well plates (VWR Plates, Fontenay-sous-Bois, France) for immunofluorescence and western blot analysis.

### 2.3. Cell Culture and Cell Transfection

Fibroblasts were cultivated in Dulbecco’s Modified Eagle’s Medium (DMEM) (Biowest, Nuaillé, France), supplemented with 15% fetal bovine serum (Eurobio, Courtaboeuf, France), 1% L-glutamine 200 mM (Life Technologies, Paisley, UK), and 1% Penicillin-Streptomycin-Amphotericin (PAA Laboratory, Pasching, Austria), in a culture flask of 25 cm^2^ (SPL Life Sciences, Gyeonggi-do, Korea), under controlled atmospheric conditions (10% O_2_, 5% CO_2_, and 85% N_2_) at 37 °C with 95% humidity. Control fibroblasts were provided by the Coriell Institute (Control 1: AG07095 and Control 2: AG09309) (Camden, New Jersey, USA). For cell transfection, fibroblasts were seeded on coverslips (Lab-tek, SPL Life Sciences, Gyeonggi-do, Korea) at a density of 2.5 × 10^4^ cells/well. Control 1 was transfected with 250 ng of plasmids containing control (pEGFP-C1) or mutated cDNA using jetPRIME^®^ transfection reagent (Polyplus Transfection, Illkirch, France), following the manufacturer’s instructions. The same reagent was used to transfect 30 nM of small interference RNA silencing specifically lamin A through interference in the 3′UTR of prelamin A transcript [12]. The efficiency of siRNA was analyzed 48 h post-transfection by immunofluorescence, using a primary antibody anti-lamin A/C (SC 6215 or SC 376248, Santa Cruz Biotechnology Inc., Santa Cruz, CA, USA) associated with a proper secondary antibody (donkey anti-goat IgG H & L, Alexa Fluor^®^ 594, ab150132, Abcam^®^, or goat anti-mouse Alexa Fluor^®^ 488, A-11001, Thermofisher, Rockford, IL 61105 USA) [13,14]. Western blotting was performed using a rabbit polyclonal anti-lamin A/C (sc-20681, Santa Cruz Biotechnology, Santa Cruz, CA, USA) and monoclonal anti-GAPDH (MAB374, Merck Millipore, Darmstadt, Germany) as loading control. Odyssey Infrared Imaging System (LI-COR Biosciences, GmbH, Bad Homburg, Germany) was used to reveal the membranes. Quantification of expression levels was performed using Image J software (National Institutes of Health, Bethesda, Maryland, MD, USA).

### 2.4. Senescence Evaluation

To evaluate the senescence rate, cells were seeded on coverslips (Lab-tek, SPL Life Sciences, Gyeonggi-do, Korea) and beta-galactosidase activity was measured using the Senescence beta-Galactosidase Staining Kit (Cell Signaling Technology^®^, WZ Leiden, The Netherlands) following the manufacturer’s instructions. To assess the replication capacity of patient and control cells, we measured 5-bromo-2′-deoxyuridine (BrdU) incorporation. To do so, cells were seeded on a 96-well plate at a density of 1.10^4^ cells/well, and BrdU was provided by the Cell proliferation ELISA; BrdU (colorimetric) Kit (Roche Applied Science, Mannheim, Germany) was added to the cell medium for a 24-h period, after which the ELISA assay was performed following the manufacturer’s instructions [13].

To quantify nuclear shape anomalies, lamin A/C immunofluorescence was performed on transfected and fixed cells as described elsewhere [15]. Phenotypes were monitored routinely by at least two independent investigators by visual inspection of fixed specimens, using an ApoTome system equipped with a 100× objective, and a charged coupled device (CCD) camera Axiocam MRm controlled by the Zen software (Carl Zeiss Microscopy, LLC, White Plains, NY, USA). Confocal images of fixed samples were performed with a LSM 800 Airyscan Axio Observer.Z1 7 confocal microscope, equipped with a 63×/1.20 W Korr UV VIS IR C-Apochromat objective, and driven by Zen 2.3 system software. Around 100 nuclei were examined for each condition; nuclear anomaly criteria consisted of aberrant nuclear lamin A staining pattern, aberrant lamin A cytoplasmic localization, and aberrant nuclei shape. The Image J 1.48v software (National Institute of Health, Bethesda, Maryland, MD, USA) was used for processing entire images before cropping to emphasize the main point of the image. Processing was limited to background subtraction and brightness/contrast adjustment.

### 2.5. RNA Sequencing and RT-qPCR

RNAseq was performed in duplicate on adipose tissue from the patients and 3 non-diabetic controls using the kit Trio RNAseq from Tecan-NuGen (Redwood City, California, CA, USA) based on human depletion on total RNA degraded. Libraries were constructed by fragmentation of the ds-cDNA, end repair, and adapter ligation. Ribodepletion was performed following the manufacturer’s instructions. The 8 indexed libraries were pooled and sequenced on an Illumina NextSeq 500 platform, using paired-end mode (2 × 75 bp reads), in order to reach 25 million reads (clusters) for each library, as an average. Total information of 70.7 Gb was obtained with a cluster passing quality control filters of 92.3%. The Gene Ontology (GO) analysis was performed with the ConsensusPathDB website (http://cpdb.molgen.mpg.de). 

Quantitative RT-PCR was performed with 200 ng of RNA, 20 U of reverse transcriptase M-MLV (Life Technologies), 50 ng of random primers, 10 mM of nucleotides, and 40 U of the ribonuclease inhibitor RNaseOUT (Life Technologies Courtaboeuf, France). Primers for quantitative PCR (RT^2^ qPCR Primer assay) were designed by Qiagen. Quantitative PCR was performed using primers labelled with fluorescent EvaGreen (Eurogentec, Angers, France) and a real-time thermocycler, Light Cycler 480 (Roche, Meylan, France). The 18s ribosomal and GAPDH RNAs were used as reference genes to normalize cycle thresholds (Ct) and to get relative quantification (ΔΔCt).

### 2.6. Statistical Analysis

For (a) cell proliferation ELISA experiments, and (b) abnormally shaped nuclei, the mean values from at least 3 independent experiments were analyzed using the Mann–Whitney test, which is a non-parametric test that compares two unpaired groups of ordinal or numerical variables. For experiments examining the proportions of cells with SA-beta-galactosidase positive staining, data from individual experiments were analyzed using the Fisher’s exact test, which is used for small sample sizes with nominal/categorical variables. Statistical calculations were performed using Prism 6.07 (GraphPad Software, San Diego, CA, USA) software. The *p*-values < 0.05 considered significant (*, *p* < 0.05; **, *p* < 0.01; ***, *p* < 0.001; ****, *p* < 0.0001).

## 3. Results

### 3.1. Patients’ Clinical Description

**Figure 1:**
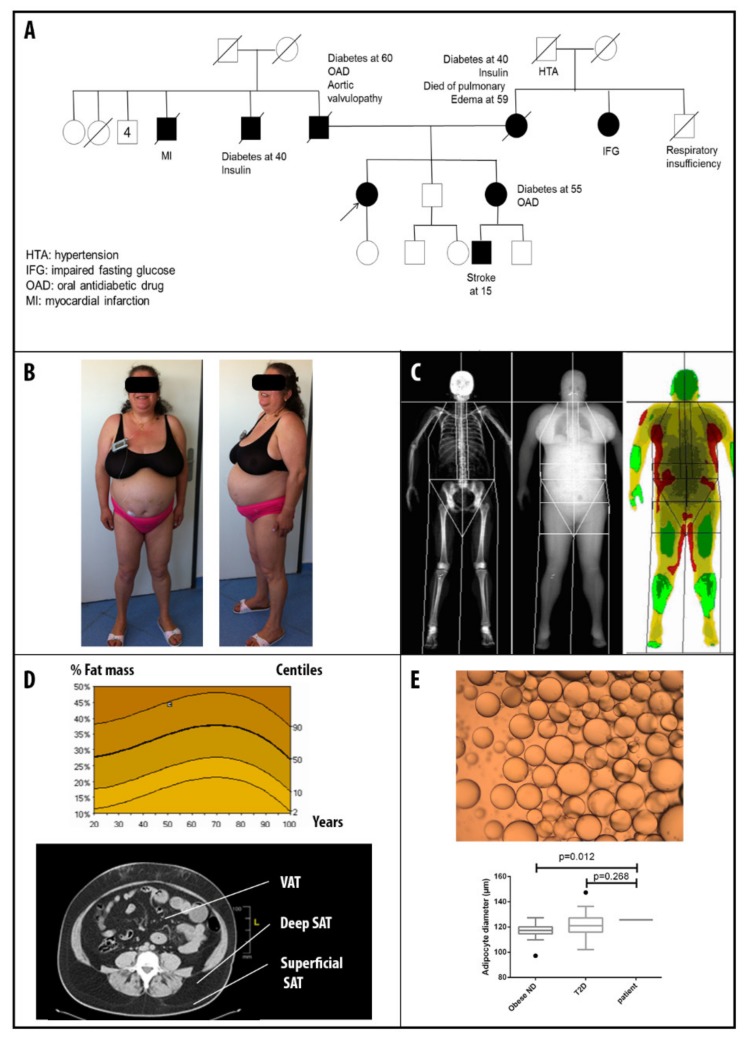
Patient 1 clinical description. (**A**) Pedigree of patient 1 family, (**B**) Photographs of patient 1 showing central/android shape obesity with fat accumulation in the facial and supraclavicular regions, and cushingoid morphotype. (**C**) Dual energy X-ray absorptiometry (DEXA) showing body fat distribution. (**D**) Percentage of fat mass compared to the reference curve and abdominal computed tomography (CT) scan confirming the presence of a small amount of subcutaneous adipose tissue (SAT) (superficial and deep), and an excessive accumulation of visceral adipose tissue (VAT). (**E**) Needle fat biopsy showing the same adipocytes diameter as a type 2 diabetic population matched for age, sex, and body mass index (BMI) and higher adipocytes diameter than obese non diabetic (ND) population matched for age, sex and BMI.

Patient 1 is a 55-year-old woman referred for early-onset insulin-resistant diabetes mellitus, diagnosed at 26, initially treated with metformin, glucagon-like peptide 1 analogs, followed by insulin (continuous subcutaneous insulin infusion, daily insulin requirement 2 UI/kg/day with HbA1c = 7.3% (56 mmol/mol)). She had a family history of diabetes mellitus (parents, sister, and one paternal uncle, Figure 1A). Her nephew had a stroke at 15 years old.

She presented high blood pressure (treated by tritherapy), combined dyslipidemia, severe hypertriglyceridemia from 4.5 to 12.5 mmol/L, and LDL-cholesterol above 2.58 mmol/L, despite high dose statins (rosuvastatin 10 mg/day), and fibrates (fenofibrate 200 mg/day), sleep apnea syndrome, and asthma. At age 42, she suffered from acute coronary syndrome, and received three stents on her left anterior descending artery. She fulfilled all the diagnosis criteria for metabolic syndrome (MS) [16].

A physical examination revealed visceral adiposity, with an accumulation of truncal fat, cushingoid morphotype with facial hirsutism, and android obesity, but no strict lipoatrophy of lower limbs (Figure 1B). Her anthropometrical features were body mass index (BMI) = 37.1 kg/m^2^, waist circumference (WC) =118 cm, waist to hip ratio (WHR) = 1.08. A dual energy X-ray absorptiometry (DEXA) revealed 44.3% of fat mass (Figure 1C and D) and an abdominal computed tomography (CT) confirmed the presence of subcutaneous adipose tissue: superficial (196 cm^2^) and deep (201 cm^2^), with excessive visceral fat accumulation (204 cm^2^) (Figure 1D). The mean adipocyte diameter obtained by the needle subcutaneous fat biopsy was 125.7 ± 32.5 µm (Figure 1E).

Cardiac investigations revealed severe coronary artery disease and concentric left ventricular hypertrophy with preserved ejection fraction (> 60%), and neither cardiac rhythm nor conduction disturbances. Hormonal investigations ruled out the diagnosis of polycystic ovarian syndrome and Cushing’s syndrome. Leptin was high at 39.3 ng/mL (reference 3.3–8.7) and adiponectin at 1.34 μg/mL (reference 3.8–11.8). Liver enzymes were in the normal range, but an abdominal echocardiography revealed diffuse hepatic steatosis.

The patient underwent bariatric surgery because of severe obesity with multiple comorbidities and a failure of lifestyle interventions. She had a sleeve gastrectomy in 2016. One year on, weight loss was 14 kilos (42 percent excess weight lost (%EWL) and the insulin doses were cut by three. Nevertheless, she put on weight again (at 24 months +6 kilos, %EWL 24%), and her insulin needs increased again. Hypertension and hypertriglyceridemia persisted after surgery and required the continuation of lipid lowering (Atorvastatine 80 mg) and antihypertensive drugs (bitherapy). Hepatic steatosis remained after the intervention, but sleep apnea syndrome improved (apnea hypopnea index decreased from 27 to 16 per hour).

**Figure 2:**
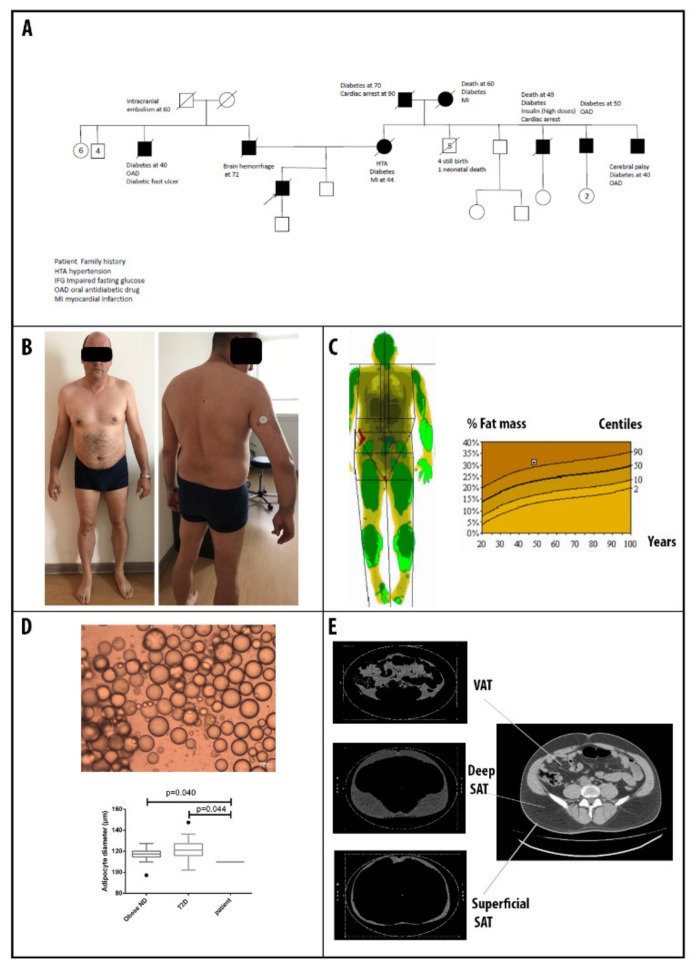
Patient 2 clinical description (**A**) Pedigree of patient 2 family. (**B**) Photographs of patient 2 showing no lower limb lipoatrophy but a high waist circumference. (**C**) DEXA showing body fat distribution and the moderate excess of fat mass (31.1%) compared to the reference curve. (**D**) Needle fat biopsy showing smaller adipocytes than a matched for age sex and BMI obese non-diabetic (ND) and a type 2 diabetic population. (**E**) Abdominal CT scan confirming the presence of an excess of deep subcutaneous fat.

Patient 2 is a 45-year-old man treated with high insulin doses (100 UI/day) for diabetes mellitus diagnosed at 34, complicated with non-proliferative retinopathy and microalbuminuria. He also suffered from hypertriglyceridemia (from 5.46 mmol/L to 13.45 mmol/L) despite high doses of statins (Rosuvastatin 10 mg/day). Leptin was measured at 8.49 ng/mL (reference 3.3–8.7). He had a family history of diabetes mellitus (Figure 2A). His mother was diabetic and died of a myocardial infarction at 44 and his father died of a brain hemorrhage at 72. On his mother’s side, diabetes mellitus was described for his grandparents and three uncles.

A physical examination revealed a BMI = 26.5 kg/m², WC = 107 cm with no lipodystrophy (Figure 2B). The patient complained of occasional muscular cramps. The percentage of fat mass was 31.1% with DEXA (Figure 2C). The mean subcutaneous adipocyte diameter was 109.9 ± 24.3 µm (Figure 2D). Abdominal CT scan revealed the presence of superficial (103 cm^2^) and deep (224 cm^2^) subcutaneous adipose tissue with excessive visceral fat accumulation (167 cm^2^) (Figure 2E). The anti -glutamic acid decarboxylase (GAD) and anti-tyrosine phosphatase-like insulinoma antigen 2 IA-2 antibodies and Maturity Onset Diabetes of the Young (MODY) testing were negatives. An abdominal echocardiography revealed liver steatosis and hepatomegaly (right hepatic arrow at 20 cm). A myocardial evaluation showed no cardiac or coronary artery disease. The exercise stress testing was normal.

### 3.2. Molecular Findings

A molecular analysis of *LMNA* showed mutations c.1892G > A p.(Gly631Asp) (rs267607648) and c.1745G > T p.(Arg582Leu), respectively, for patients 1 and 2 as the only potentially pathogenic variations in a panel of 82 genes involved in premature aging, lipodystrophy syndromes, and diabetes (list available on request). Both mutations are very rare (allelic frequency in GnomAD: 8.037 × 10^−6^ and 1.095 × 10^−5^ https://gnomad.broadinstitute.org/) with no clinical data available, and are predicted to be pathogenic (ACMG class 5), or likely pathogenic (ACMG class 4), by several bioinformatics tools (http://umd-predictor.eu/, https://varsome.com/) [17,18].

### 3.3. Patient Fibroblasts Functional Analysis

**Figure 3:**
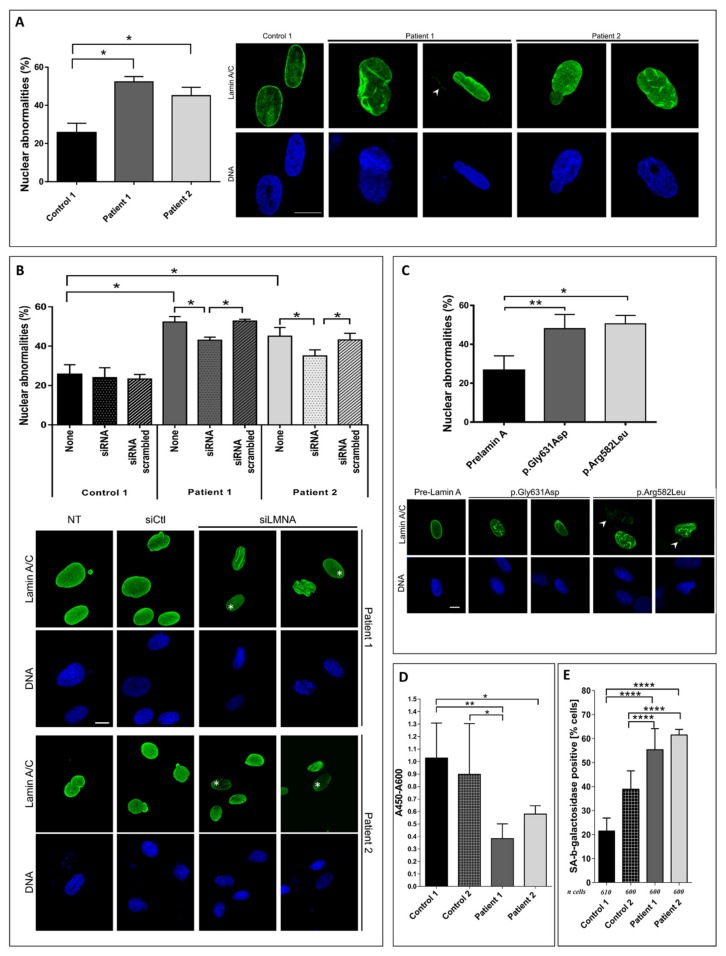
Nuclear shape anomalies and senescence studies. (**A**) Percent of nuclear shape anomalies counted by immunofluorescence in control (in black), and patient (in grey) cells (left panel), and representative pictures of control and patient cells after staining with DAPI and anti-lamin A fluorescent antibody (right panel). (**B**) Percent of nuclear shape anomalies after transfection in control and patient fibroblasts with a siRNA targeting 3′UTR of *LMNA* (upper panel); representative pictures of patient cells showing improved nuclear shape after siRNA transfection (lower panel); * represents low staining in nuclei with normal shape. (**C**) Percent of nuclear shape anomalies after transfection of control fibroblasts with plasmids expressing either wild type prelamin A (in black) or mutants prelamin A (in grey) tagged with GFP at the N-terminal end (left panel) and representative pictures of each conditions (right panel). (**D**) BrdU incorporation of control and patient cells. (**E**) Senescence associated to beta-galactosidase production. In these experiments, controls correspond to fibroblasts from healthy donors. White arrows on the pictures show cytoplasmic staining and scale bars 5 μm. The number of independent experiments was between three and seven. Asterisks correspond to significant *p*-values: * *p* < 0.05, ** *p* < 0.01, **** *p* < 0.0001.

After immunostaining with lamin A/C antibodies, we observed for both patient cells, an altered lamin A/C staining with reduced signal, and heterogeneous staining with aggregates. Nuclear shape anomalies were quantified in primary fibroblasts at passage 16, and showed a 2-fold increase of the percentage of nuclear anomalies, such as misshapen nuclei or nuclear membrane blebs in patient cells when compared to two control cells (Figure 3A).

To confirm that mutant lamin A expression is responsible for increased abnormal nuclear shape, we first evaluated the effect of lamin A down-expression on the nuclear morphology of patient fibroblasts. We transfected the patient cells with a siRNA specifically targeting the 3′UTR of *LMNA* transcripts, which induces a significant decrease of lamin A level expression [13,19]. While the *LMNA*-specific siRNA did not induce a significant modification of the proportion of abnormal nuclei in the control cells, it did lead to a significant improvement of nuclear phenotypes in the patient cells (Figure 3B). Indeed, under siRNA treatment, the lamin A expression level was reduced by a third, leading to a 10–15% nuclear anomalies reduction in patient fibroblasts. This reduction was moderate but significant as it was observed in five independent experiments. Secondly, in another experiment, we evaluated the effect of *LMNA* mutant’s expression on nuclear morphology, by transient transfection of mutation-expressing plasmids in wild type cells. To do so, we transfected plasmids expressing wild type or *LMNA* mutant cDNA carrying either patient 1 or 2 mutations, and tagged with GFP at the N-terminal end. A significant higher level of nuclear abnormalities was observed in cells expressing *LMNA* mutants compared to control cells transfected with wild type lamin A (Figure 3C). Thus, while the depletion of lamin A in patient cells significantly improved nuclear abnormalities, the expression of lamin A mutants mimics these phenotypes, suggesting that mutants are at least partially responsible for nuclear deformations observed in patient fibroblasts.

Next, we assessed senescence by two different tests: first, by evaluating the replication profile of each patient’s cells through the estimation of BrdU incorporation. As shown in Figure 3D, BrdU incorporation was significantly lower in patient 1 cells compared to both controls, and in patient 2 cells only when compared to control 1. In a second set of experiments, the production of beta-galactosidase was evaluated as a common marker of cellular senescence. The results showed a significantly higher rate of beta-galactosidase staining, indicating increased senescence for both patient cells (Figure 3E).

### 3.4. RNA Sequencing Analysis in Adipose Tissue

**Figure 4:**
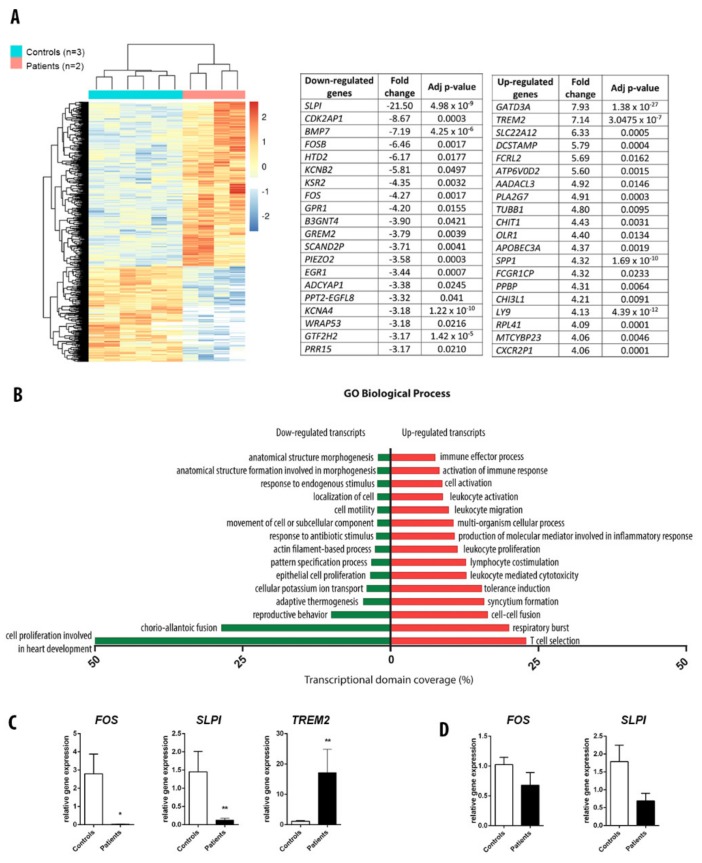
RNAseq on whole adipose tissue biopsies. (**A**) Heat-map and top-20 of deregulated genes in the two patients with *LMNA* mutations compared to three controls paired for age and sex. (**B**) GO analysis showing the main pathways deregulated in patients with lamin A mutation. (**C**) Validation by RT-qPCR of three regulated genes in controls and patients (*TREM2, SLPI, FOS*) in adipose tissue, * *p* < 0.05 vs. controls; ** *p* < 0.01 vs. controls. (**D**) RT-qPCR of SLPI and FOS in fibroblasts. *TREM2* was not expressed in fibroblasts.

We further compared the transcriptome of the whole subcutaneous adipose tissue of the two patients and three controls paired for age and sex using RNA sequencing. The genes presenting the most important fold change in expression are presented in Figure 4A. Data analysis showed upregulation of genes implicated in immunity, such as *TREM2*, *DCSTAMP*, *FCRL2,* and down-regulation of genes involved in the development and cell differentiation, such as *SLPI*, *CDK2AP1*, *BMP7*, *FOS*. Interestingly, two genes (one down- (*B3GNT4)* and one upregulated (*SLC22A12)*) were implicated in uric acid homeostasis, and two down-regulated genes were found to be implicated in glucose metabolism (*KSR2* and *GPR1*). Remarkably, GO analysis identified that genes deregulated in patients were implicated mainly in immunity and development pathways, such as T cell selection, leucocyte mediated cytotoxicity, lymphocyte co-stimulation, migration and activation, inflammatory response, and cell proliferation (Figure 4B). Separate analysis, one-to-one patient compared to controls, showed in the top-20 deregulated genes, two genes similarly deregulated in the two patients. One is *SPLI*, which has been validated by q-PCR, the other is *GATD3A,* a potential mitochondrial protein, which function remains to be fully determined (data not shown). Differentially expressed genes (*SLPI, TREM2, FOS*) were validated by RT-qPCR to confirm the RNAseq results (Figure 4C). By contrast, these genes were not differentially expressed in fibroblasts of patients and controls, and as expected, *TREM2* was not expressed in fibroblasts (Figure 4D).

## 4. Discussion

We have shown two rare missense mutations in *LMNA;* both were associated with severe metabolic phenotypes, resistance to bariatric surgery-induced weight loss in patient 1, alterations in the shape of nuclei, increased beta-galactosidase staining and modifications of gene expression patterns in subcutaneous adipose tissue and fibroblasts. As suggested by Caron et al. [20] these cellular and phenotypic alterations could be suggestive of metabolic laminopathy.

Both mutations are located on exon 11 corresponding to the C-tail specific domain of lamin A, consequently, lamin C is not affected, and observed alterations can be attributed to the presence of mutated lamin A only. Here, the phenotype induced by the mutation p.(Gly631Asp) (patient 1) seem clinically more severe than the one associated with mutation p.(Arg582Leu) (patient 2). This could be explained either because of the mutation nature in itself or the patient’s sex, as women are generally more affected in genetic lipodystrophy than men [10]. Remarkably, patient 1 did not benefit from the expected weight loss after bariatric surgery (<50% EWL) and the metabolic abnormalities (hypertension, dyslipidemia, and insulin-requiring type 2 diabetes) remained, despite an important caloric restriction induced by the sleeve gastrectomy, showing that her metabolic phenotype was not only due to an environmental Western lifestyle. The effect of bariatric surgery on metabolic laminopathy had never been reported to date. This should encourage researchers to try to understand the role of molecules derived from fat accumulation to the onset and progression of the disease.

Only a few cases of MS linked to *LMNA* mutations have been reported up to now, suggesting that this condition, given the high proportion of patients with MS or metabolic abnormalities with early onset, is under-diagnosed [10,13,14,21]. Indeed, a recent study based on the analysis of variant frequencies in several public databases gathering data on 60, 706 unrelated individuals, revealed an unexpected high number of *LMNA* variants (*n* = 132), including novel variants predicted to perturb lamin A assembly or function. Among them, the variant p.Gly602Ser was identified as a potential risk factor for type 2 diabetes mellitus in African Americans [22].

It is particularly important to pay attention to metabolic derangements induced by *LMNA* mutations because specific clinical follow-up and therapeutic approaches can be set up for these patients, as mentioned in the multi-society practice guidelines published in 2016 [3]. This is the first work reporting the poor effect of a restrictive bariatric surgery on a patient with *LMNA* mutation.

Furthermore, an anticipation of metabolic complications over three generations of patients carrying mutated *LMNA* has been described, such as hypertriglyceridemia, which appeared 15 years earlier in the second generation, highlighting the importance of pre-symptomatic genetic diagnosis [23]. Finally, one important finding of this work was the persistence of subcutaneous adipose tissue in the described patients, which was consistent with the measurable serum leptin level that could make the diagnosis of laminopathy harder, but also extend the pattern of *LMNA* associated phenotypes to cardio-metabolic syndrome without subcutaneous lipoatrophy.

We observed a slight but constant decrease in the nuclear abnormalities after siRNA treatments, over several independent experiments. Moderate reduction of mutated (and WT) lamin A/C improved to some extent the fibroblast cellular phenotype, possibly due to the lowering of prelamin A levels. Using experiments commonly performed to study senescence in vitro, we showed that patient cells exhibit premature senescence, but to a lesser extent than the senescence induced by the progeria mutation, or the canonical FPLD2 mutation R482W. Indeed, when compared to control cells issued from a person with comparable age, we observed no significant differences in population doubling level (data not shown), and the modifications of the beta-galactosidase and BrdU tests results, although significant, were moderate. These results are in accordance with the clinical phenotypes described here, which are milder than progeria, or even typical FPLD2.

In previous studies, lamin A/C has been shown to mediate the activation of adipose tissue macrophage inflammation by regulating the proinflammatory factors, such as NF-κB, or TNFα, contributing to the development of insulin resistance [24]. In addition, the depletion of lamin A/C in macrophages or myeloid cells reduced the lipopolysaccharide LPS-induced expression of proinflammatory genes and adipose tissue inflammation [24]. Interestingly, in our study, RNA sequencing of the whole subcutaneous adipose tissue of patients revealed an upregulation of genes involved in immunity (Figure 4B). More specifically, we showed that *TREM2* was upregulated in the adipose tissue of both patients carrying lamin A mutations with a fold change of 7 (*p* < 0.001). *TREM2* gene encodes a type I transmembrane protein that is a member of the immunoglobulin (Ig) receptor superfamily and was shown to be a major macrophage sensor of extracellular lipids. Furthermore, we observed a drastic decrease of *SLPI* in the adipose tissue of the two patients. *SLPI* gene encodes a secretory leukocyte protease inhibitor, which is an important anti-inflammatory adipokine that counteracts adipose tissue inflammation and enhances browning of adipose tissue [25].

Recent advances on pathological mechanisms involved in progeroid or tissue specific laminopathies, report the activation of the AKT/mTOR pathway, ultimately impairing the autophagic activity [26]. In our experiments using RNAseq, we did not notice any alteration in the expression level of genes involved in this pathway.

## 5. Conclusions

Our work has shown that two heterozygous rare missense mutations in *LMNA* are associated with severe metabolic alterations, such as hypertriglyceridemia and insulin resistance, and are associated with premature senescence at the cellular level. Altogether, our results confirm that the pattern of *LMNA* associated phenotypes should be extended to cardio-metabolic phenotypes without subcutaneous lipoatrophy.

Future studies are needed to define which patients should be genetically screened, as it is likely that this condition is under-diagnosed, given the high prevalence of metabolic diseases worldwide. Further advances may provide the insights necessary to understand the many mechanisms and pathways by which A-type lamins influence metabolism.
